# Extracellular vesicles derived from fibroblasts induced with or without high glucose exert opposite effects on wound healing and angiogenesis

**DOI:** 10.3389/fsurg.2022.1065172

**Published:** 2022-11-28

**Authors:** Xiaowei Bian, Bingmin Li, Haowen Tang, Qiankun Li, Wenzhi Hu, Qian Wei, Kui Ma, Yuguang Yang, Haihong Li, Xiaobing Fu, Cuiping Zhang

**Affiliations:** ^1^ Research Center for Tissue Repair and Regeneration Affiliated to the Medical Innovation Research Division of Chinese PLA General Hospital, Beijing, China; ^2^ Department of Dermatology, Fourth Medical Center of Chinese PLA General Hospital, Beijing, China; ^3^ Department of Hepatobiliary Surgery, First Medical Center of Chinese PLA General Hospital, Beijing, China; ^4^ Research Unit of Trauma Care, Tissue Repair and Regeneration, Chinese Academy of Medical Sciences, Beijing, China; ^5^ Beijing Key Research Laboratory of Skin Injury, Repair and Regeneration, Beijing, China; ^6^ Department of Wound Repair, Institute of Wound Repair and Regeneration Medicine, Southern University of Science and Technology Hospital, Southern University of Science and Technology School of Medicine, Shenzhen, China

**Keywords:** extracellular vesicles, high glucose, fibroblasts, endothelial cells, angiogenesis, wound healing

## Abstract

**Background:**

Communication between fibroblasts and endothelial cells is essential for skin wound repair and regeneration. Extracellular vesicles (EVs) are crucial for intracellular communication by transporting active molecules. However, whether EVs derived from diabetic fibroblasts can perform the nomal communication function is unclear. Here, we compared the effects of EVs from human skin fibroblasts (HSFs) induced with or without HG on the angiogenic function of endothelial cells and wound healing.

**Methods:**

We first collected EVs from HSFs cultured with normal glucose concentration (NG-EVs) or with HG concentration (HG-EVs) and applied them to treat human umbilical vein endothelial cells (HUVECs). The cells were divided into three groups: control group, NG-EVs group, and HG-EVs group. We then examined the proliferation, migration, apoptosis, and tube formation of HUVECs. To illustrate the mechanism, the expression of β-catenin, GSK-3β, and p-GSK-3β was detected by western-blot. Finally, NG-EVs or HG-EVs were used to treat the wounds of mice to determine their role in wound closure.

**Results:**

By DNA content detection, Annexin V/PI staining, and EdU staining, we found that NG-EVs promoted HUVEC proliferation, while HG-EVs exhibited an opposite effect (*p* < 0.05). Scratch assay and tube formation assay demonstrated that NG-EV promoted angiogenesis *in vitro*, while HG-EVs showed negative impact (*p* < 0.05). The expressions of β-catenin and p-GSK-3β in HUVECs were enhanced by NG-EVs and decreased by HG-EVs (*p* < 0.05). Additionally, the *in vivo* experiment demonstrated that NG-EVs effectively promoted wound healing by locally enhancing blood supply and angiogenesis. In contrast, HG-EVs leaded to delayed wound closure and reduced blood supply and angiogenesis (*p* < 0.05).

**Conclusion:**

NG-EVs and HG-EVs exert opposite effects on wound healing and angiogenesis possibly by regulating GSK-3β/β-catenin signaling pathway. This research may provide a new treatment strategy for wound healing and illustrate the mechanism for impaired angiogenesis in diabetics.

## Introduction

As the population ages and health-care costs gallop ahead, the incidence of diabetic wounds has been rising, which burdens both individual and our society. Angiogenesis means the development of new capillaries from preexisting vessels, which plays a critical role in the wound healing process. After injury, the homeostasis is interrupted, leading to a hypoxic state. Numerous proangiogenic factors are produced under hypoxia circumstances. These factors, the most notable of which is VEGF, activate the endothelial cells, stimulate capillaries to form nascent immature loops and branches to provide oxygen and nutrients to the wound site (1, 2). Communication between fibroblasts and endothelial cells is essential for skin wound repair and regeneration. Fibroblasts can secrete angiogenic growth factors such as bFGF and VEGF, which are potent mitogens and crucial for angiogenesis (3).

Extracellular vesicles (EVs), defined as 50–150-nm-sized vesicles, are membrane-enclosed and secreted by many types of cells *in vitro* and *in vivo*. As natural carriers, EVs can transfer endogenous bioactive molecules, such as RNA, proteins and lipids to recipient cells, thereby playing an indispensable role in cell-to-cell communication (4–6). Furthermore, the composition of EVs differs according to their origin, and thus, the carried information is also different (7). Additionally, the biological characteristics and functions of EVs derived from the same donor cells under different physiological and pathological conditions may also be different.

Substantial evidence suggests that high blood glucose level results in fibroblast dysfunction. The chronic hyperglycemic state enhances the formation of reactive oxygen species (ROS). The increased ROS production accelerates telomere shortening, induces the DNA damage response, thereby altering cellular functions (8). Li et al. have reported that hyperglycemic condition impairs the biological function of fibroblast by activation of p21 and p16 in a reactive oxygen species (ROS)-dependent manner (9). Under normal physiological condition, EVs derived from dermal fibroblasts promote diabetic cutaneous wound healing through the Akt/β-catenin pathway (10). Another report showed that EVs isolated from mouse embryonic fibroblasts augmented the effect of VEGF, promoting angiogenesis in the wound area (11). Gangadaran et al. (12) have already confirmed the angiogenic cargoes in human fibroblast-derived EVs. However, whether EVs derived from diabetic fibroblasts can perform the normal function during angiogenesis is unclear.

In the current study, we isolated EVs from HSFs cultured with normal glucose concentration (NG-EVs) or with HG concentration (HG-EVs) and applied them to treat HUVECs and the wounds of mice. We found NG-EVs and HG-EVs performed the opposite effects on wound healing and angiogenesis possibly by regulating GSK-3β/β-catenin signaling pathway.

## Subjects and methods

### Isolation and characteristics of EVs

Fibroblasts were derived from human foreskin explants and cultured in Dulbecco's Modified Eagle's Medium (DMEM, Gibco, USA, 11885092), The concentration of glucose in the culture medium was 5.5 mmol/L in normal glucose (NG) group, and 30 mmol/L in high glucose (HG) group. After 5 days, the cultured medium was collected every 48 h from passage 3 to passage 7. The supernatants of these cells were mixed together. EVs were collected from supernatant by a series of ultra-high-speed centrifugation procedures according to the steps reported in literatures (13).

The morphology of NG-EVs and HG-EVs was observed by transmission electron microscope (TEM) (Hitachi, Japan) and the particle size was measured by nanoparticle tracking analyzer (Particle Metrix GmbH, Germany). Expressions of EV positive markers CD63 (abcam, ab68418), TSG101 (abcam, ab133586), and negtive marker calnexin (abcam, ab133615) were analyzed by Western blot. A brief step of uptake assay is as follows. The membrane of EVs was labeled with PKH67 (green). HUVECs were counterstained by actin (cytoskeleton, red) and DAPI (cell nucleus, blue). The internalization process was observed under confocal microscope (Leica, Germany).

### Cell culture

HUVECs were purchased from the Chinese Academy of Sciences (Shanghai, China) and cultured in low glucose Dulbecco's modified Eagle's medium (DMEM, Gibco, USA, 11885092) supplemented with 10% fetal bovine serum (Gibco, USA, 16140089). Our previous work had demonstrated that EVs at the concentration of 5 × 10^11^ particles/ml achieved a better effect *in vitro* (13). Therefore, we used the 5 × 10^11^ particles/ml EV dissolution in the *in vitro* experiments.

### Cell proliferation assay

The EdU proliferation assay was conducted to detect DNA synthesis. Briefly, cells were seeded in 24-well plates at the density of 1 × 10^4^ cells per well. After 24 h, the cells were incubated with 5-ethynyl-2'-deoxyuridine (EdU) (BeyoClickEdU 488, Beyotime, C0071l) for 2 h. After fixed, the labeled cells were incubated in the click addictive solution in a dark humidifier for 30 min. The labeled cells glow green and the nuclei stained by Hoechst 33342 glow blue. Images were taken by fluorescence microscope (Olympus U-RFL-T, Tokyo, Japan).

### Cell cycle

A DNA Content Quantitation Assay kit (Solarbio, CA1510) was employed to detect cell cycle. Briefly, the cells were cultured in different medium for 72 h and fixed in 70% ethanol overnight. The suspensions were digested with 100 μl RNase A (30 min, 37 °C), and subsequently labeled by 400 μl propidium iodide (PI) (30 min, 4 °C, preserved in dark place). The PI fluorescence of each group was measured by flow cytometer (BD FACS Calibur TM, Becton-Dickinson, USA), and the percentage of cells in each phase was calculated.

### Cell apoptosis

After cultured in specific media for 3 days, HUVECs were collected and suspended in PBS. AnnexinV Alexa Fluor488/PI kit (Solarbio, CA1040) was employed to detect cell apoptosis. Firstly, the cell density was adjusted by binding buffer to about 1–5 × 10^6^/ml. Next, 100 µl cell suspension was incubated with 5 µl Annexin V/Alexa Fluor 488 in a 5 ml flow cytometry tube for 5 min, and then added 10 µl 20 µg/ml propidium iodide (PI) and 400 µl PBS. The apoptosis rate of HUVECs was detected immediately by flow cytometry (BD biosciences, USA).

### Scratch assay

The migration property of HUVECs was evaluated by scratch assay. HUVECs were inoculated in six-well plates and cultured in regular medium. When reached 90% confluence, the cells were scraped with a pipette tip. After washed by PBS for three times, the cells were cultured in DMEM medium contained NG-EVs or HG-EVs. Photographs were taken under a phase-contrast microscope at 0 and 24 h respectively. The capacity of migration was evaluated by comparing the percentage of wound closure area, which was calculated as (initial scratch area—healed scratch area)/initial scratch area × 100%.

### Tube formation

HUVECs were seeded into a μ-slide angiogenesis glass bottom (ibidi, 81507) and treated with different mediums. After incubated for 8 h, at least three randomly chosen fields were examined under microscope. The total branching points and total tube length were recorded to reflect the tube formation capability.

### Western blot analysis

Protein of cells cultured in specific media was extracted by RIPA buffer (Invitrogen), separated by 12% SDS-PAGE gels, and transferred onto PVDF membrane. The blots were blocked with 5% non-fat dried milk, incubated with primary antibodies including β-catenin (Beyotime, AC106), GSK-3β (Beyotime, AG751), p-GSK-3β (Beyotime, AF1531), and GAPDH (Beyotime, AF0006) at 4 °C overnight, and with secondary antibodies at 37 °C for 2 h. The immunoreactive bonds were developed by ECL kit (solarbio, PE0010) and exposured by the ImageQuant LAS 4000 system.

### Animal study

The study protocol was approved by the Ethics Committees of PLA General Hospital. Sixty C57BL/6J mice were involved in this experiment. The mice were randomly divided into NG-EV group, HG-EV group and control group. After anesthetized with 4% chloral hydrate, a 1 cm diameter full-thickness excision was performed on the back of each mouse. The NG-EV group and HG-EV group were injected with 100 μl EVs (the concentration of EVs was about 5 × 10^11^ particles/ml) around the wound area at 4 sites at day 0, 2, 4, and 6 post operation, while the control group was treated with 100 μl PBS. At 2, 4, 6, and 8 d after surgery, we took photos to document the wound closure process. The wound tissues were excised for histological analysis and the percentage of wound closure was calculated as: % of wound closure = 100 × (original wound area—actual wound area)/original wound area. At day 4 after surgery, the blood perfusion at the wound sites was detected by a laser Doppler perfusion imaging system.

### Statistical analysis

The data were reported as the means ± SD, and all the experiments were conducted at least three times. Statistical analysis was performed using one-way analysis of variance (ANOVA) for comparison of group means. Two-way ANOVA was employed for comparison among groups at different time points. *p* < 0.05 was considered statistically significant.

## Results

### Characteristics of EVs

**Figure 1:**
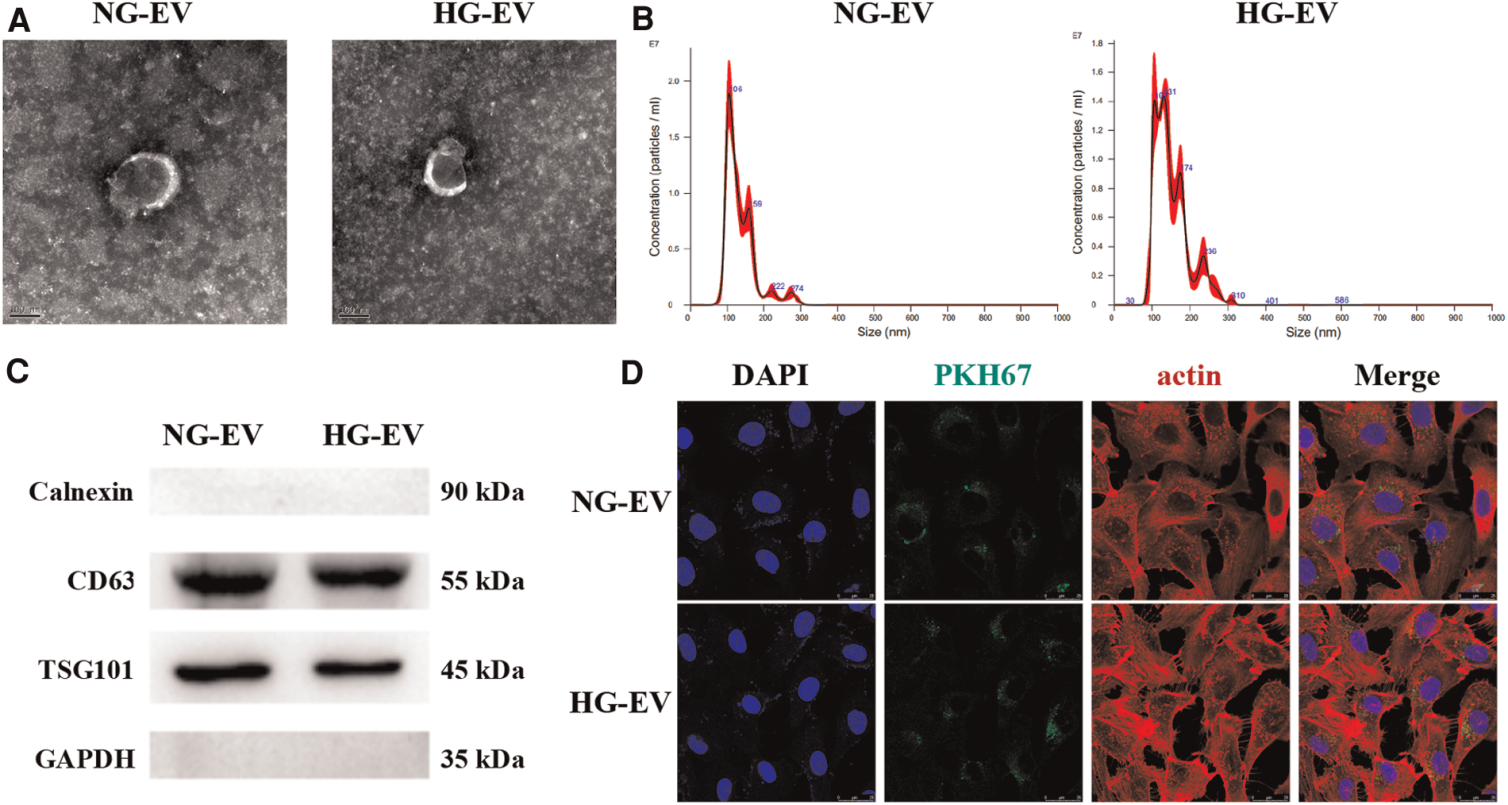
Characteristics of EVs. (**A**) Ultrastructure of EVs, scale bar = 100 nm. (**B**) Particle size distribution measured by nanoparticle tracking analyzer. (**C**) The expression level of CD81, TSG101 and Calnexin measured by western-blot. (**D**) Uptake assay. The membrane of EVs were labeled with PKH67 (green). HUVECs cytoskeleton was counterstained by actin (red) and nucleus was labeled by DAPI (blue).

The characteristics of EVs were determined by TEM, NTA and Western blotting. The ultrastructure of EVs was presented in Figure 1A, which showed a typical cup shape and a smooth double-layer structure. The diameters of particles were calculated by nanoparticle tracking analyzer (NTA) (Figure 1B). In NG-EV group, the mean diameter of particles was 135.7 nm and the peak particle size was 105.7 nm. In HG-EV group, the mean diameter of particles was 149.0 nm and the peak particle size was 130.8 nm, which was significantly larger than that in NG-EV group (*p* < 0.05). Although the morphological characteristics in both groups were in accordance with the descriptions of EVs in previous studies (14), the possible mechanism of the changes in particle size was presumably caused by encapsulated some metabolites, such as advanced glycation end products (AGEs). The positive markers of EVs including CD63 and TSG101 were detected by Western blot and the negative marker calnexin was not detectable in both group (Figure 1C). Uptake assay revealed that after 12 h, the EVs in both groups (labeled with PKH-67, green fluorescence) could be transferred into HUVECs (cytoskeleton of HUVECs was labeled by actin presenting red fluorescence). As shown in Figure 1D, there was no difference between two groups in the uptake rate, which makes sure that the difference between two groups in biological functions was caused by the different compounds inside the vesicles.

### Effects of NG-EVs and HG-EVs on the proliferation of HUVECs

**Figure 2:**
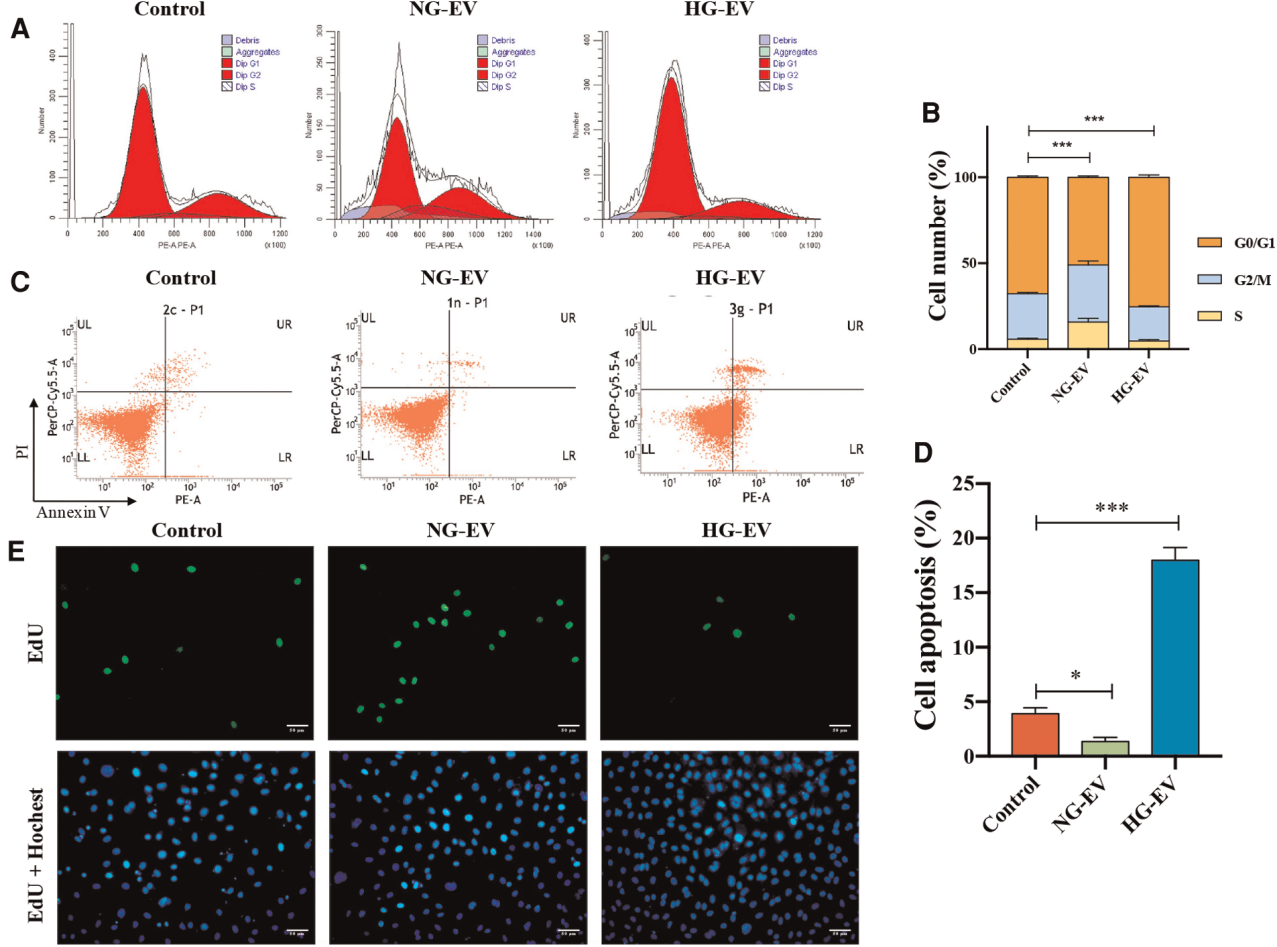
The effects of EVs on HUVECs proliferation. (**A**) Flow cytometric analysis of cell cycle. (**B**) Flow cytometric analysis of cell apoptosis. (**C**) The percentage of HUVECs in the G0/G1, S and G2/M phases, respectively (*n* = 5). (**D**) The statistical analysis of the percentage of EdU positive cells (*n* = 5). (**E**) EdU positive cells (green) and nuclei (blue) in each group.

To investigate the effect of EVs on cell cycle, the DNA content was determined by flow cytometry and the cell subpopulations (G0/G1, S and G2/M) were calculated. In NG-EV group, the percentage of G_0_/G_1_ subpopulation was decreased and accordingly the percentage of S and G_2_/M subpopulations was increased. In contrast, HG-EVs has an opposite effect on HUVECs (Figures 2A,B). This result indicated that NG-EVs promoted HUVEC proliferation while HG-EVs retarded the process. Compared with control group, the apoptosis rate was down-regulated in NG-EV group, while the apoptosis rate in HG-EV group was markedly up-regulated and came up to 16.8% (*p* < 0.01) (Figures 2C,D). These results were further confirmed by EdU staining. As shown in Figure 2E, the percentage of EdU-positive cells significantly increased in NG-EV group, while decreased in HG-EV group. The results above demonstrated that NG-EVs promoted HUVEC proliferation, while HG-EVs exhibited an opposite effect.

### Effects of NG-EVs and HG-EVs on the migration and tube formation of HUVECs

**Figure 3:**
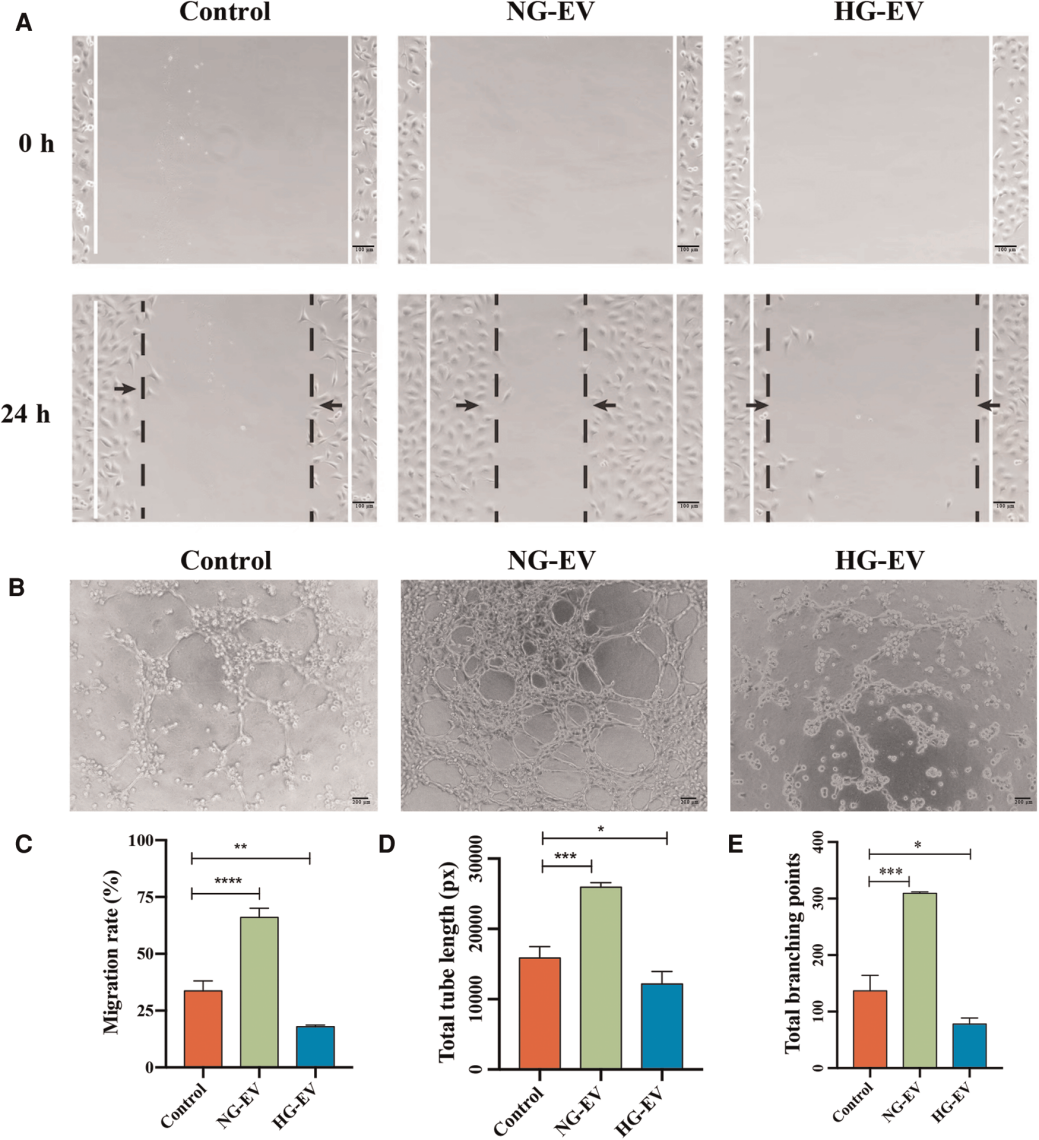
The effects of EVs on HUVECs migration and tube formation. (**A**) 0 and 24 h after scratch. (**B**) The images of tube formation of HUVECs in each group. (**C**) The statistical analysis of wound closure area (*n* = 5). (**D,E**) Quantitative analysis of tube formation ability.

Angiogenesis plays a critical role in wound healing by providing nutrition and oxygen and removing cell waste. The abilities of migration and tube formation of HUVECs are the important indicators for angiogenesis. Scratch assay was performed to assess the effect of EVs on the migration ability of HUVECs. As shown in Figures 3A,C, the migration area was significantly increased in NG-EV group while decreased in HG-EV group, which demonstrated that NG-EVs enhanced HUVEC motility while HG-EVs impaired the migratory ability. Compared with control group, the number of capillary-like structures was increased in NG-EV group while decreased in HG-EV group. Quantitative measurements also revealed a similar trend, which showed that the total branch points and total branching length were significantly increased in NG-EV group, while decreased in HG-EV group (Figures 3B,D,E). These results indicated that NG-EV promoted angiogenesis *in vitro*, while HG-EVs showed negative impact.

### Effects of NG-EVs and HG-EVs on activation of GSK-3β/β-catenin pathway

**Figure 4:**
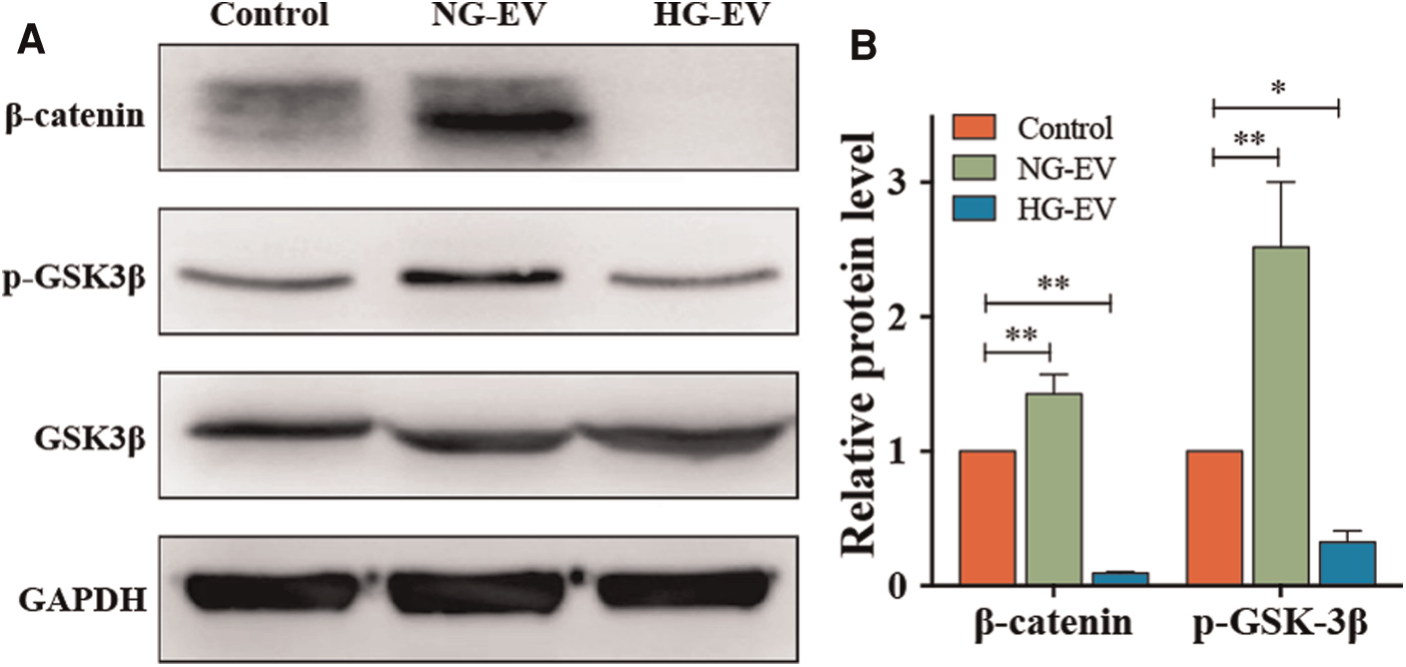
Evs regulate HUVECs functions by regulating GSK-3β/β-catenin signaling pathway. (**A**) The expression level of β-catenin, p-GSK3β and GSK3β in each group. (**B**) The relative protein level in each group. The result was normalized to GAPDH expression.

To illustrate the regulatory mechanism for the effects of NG-EVs and HG-EVs on HUVECs, the expression of GSK-3β, p-GSK-3β and β-catenin was detected by Western blot. The results indicated that NG-EVs up-regulated the expression of p-GSK-3β and β-catenin, while HG-EVs significantly down-regulated the GSK-3β/β-catenin pathway (*p* < 0.05) (Figure 4). As is known, the activation of GSK-3β/β-catenin pathway can improve the biological functions of HUVECs (15). Therefore, the opposite effects of NG-EVs and HG-EVs on biological functions of HUVECs might due to the different impact of EVs on GSK-3β/β-catenin signaling pathway.

### Effects of NG-EVs and HG-EVs on cutaneous wound healing and blood perfusion

**Figure 5:**
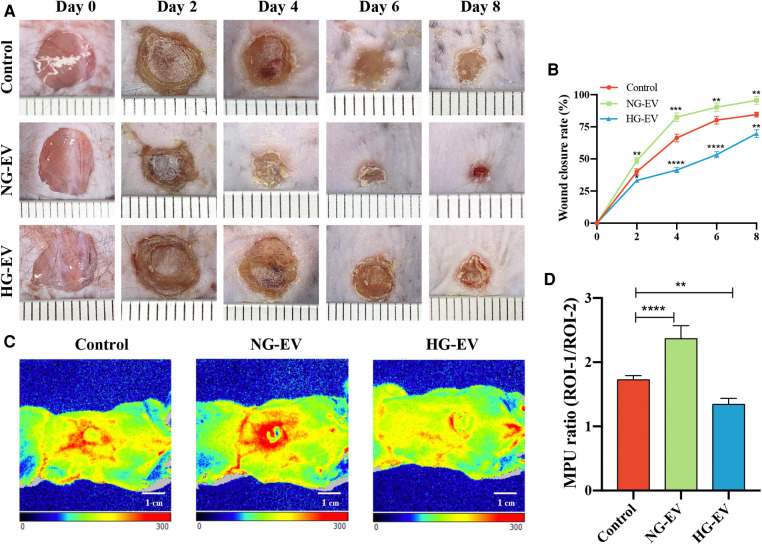
The impact of EVs on cutaneous wound healing and blood perfusion. (**A**) Healing progression of the wound from day 0 to day 8. (**B**) The statistical analysis of wound closure area in each group (*n* = 5). (**C**) The blood flow at the wound site in each group that was evaluated by laser Doppler perfusion imaging system. (**D**) Quantitative perfusion intensity of the wound area in the three groups.

A mouse full-thickness skin wound model was employed to evaluate the effects of EVs on wound healing *in vivo*. Compared to the control group, the mean wound area of NG-EV group was significantly smaller (*p* < 0.01), while HG-EV group exhibited a delayed wound closure process (Figures 5A,B). At day 4, the blood flow at wound sites was evaluated. The red area and the density of flux images represent the blood perfusion level around the wound site. As shown in Figures 5C,D, NG-EVs significantly improved the blood perfusion level, while HG-EVs retarded blood circulation. These results demonstrated that NG-EVs effectively promoted blood perfusion and wound healing, but HG-EVs leaded to delayed wound closure and reduced blood supply and angiogenesis.

## Discussion

In this study, we reported that NG-EVs promoted wound healing as well as blood perfusion, while HG-EVs have an opposite effect *in vivo*. In vitro studies showed that: (a) NG-EVs promoted HUVEC proliferation, while HG-EVs exhibited an opposite effect; (b) NG-EVs promoted angiogenesis by facilitating the migration and tube formation ability of HUVEC, while HG-EVs showed negative impact; (c) The opposite effects of NG-EVs and HG-EVs on HUVECs biological functions might due to the different impact of EVs on the GSK-3β/β-catenin signaling pathway.

Wound healing requires the participation of a variety of cells, cytokines, and nutrients, as well as adequate oxygen supply. As the medium of substance exchange, blood vessels play a vital role in the process of wound repair. Therefore, promotion of angiogenesis is necessary for early restoration of blood supply in injured tissues (16). The process of angiogenesis is well-orchestrated and regulated by many kinds of cytokines, such as fibroblast growth factor (FGF), vascular endothelial growth factor (VEGF), transforming growth factor (TGF-β1), hypoxia inducible factor (HIF-1α), and so on. Recent studies indicate that fibroblasts regulate angiogenesis by secreting angiogenic cytokines and mobilizing the sprouting vessels in wound healing (17). Many researchers demonstrated that hyperglycemic environment reduced angiogenesis due to the injured function of endothelial cells (18, 19). Besides, hyperglycemic environment also impairs fibroblast function. Our previous work demonstrated that high glucose environment decreased the proliferation and migration abilities and induced premature senescence of fibroblasts by up-regulating p53/p21/p16 signaling pathway (8). The accumulation of advanced glycation end products (AGEs) is thought to be the important factor for cell malfunction and senescence (20). Recently, the EVs derived from fibroblasts under normal condition can promote angiogenesis, thereby accelerating wound healing. However, whether EVs derived from diabetic fibroblasts can promote angiogenesis needs to be investigated.

In the present study, we found that NG-EVs promoted the proliferation, migration, and tube formation of HUVECs *in vitro* and accelerated wound healing and blood supply *in vivo*, while HG-EVs exhibited the opposite effects. Further study showed that NG-EVs up-regulated the expression of p-GSK-3β and β-catenin, while HG-EVs down-regulated the GSK-3β/β-catenin pathway. The possible reason might be related to the different composition in NG-EVs and HG-EVs. NG-EVs may contain angiogenic cytokines such as bFGF and VEGF, while HG-EVs may contain metabolites such as AGEs, which leads to HUVEC damage. In line with our study, Newman et al. reported that the fibroblast-derived bioactive substances, such as collagen I, procollagen C endopeptidase enhancer 1 and TGF-β-induced protein ig-h3 etc. were necessary for lumen formation and angiogenesis (21). Besides, Zhang et al. reported that AGEs increase ROS formation and lead to vascular endothelial dysfunction (22). Rezaie and Shariatzadeh et al. also proved that serum from patients with type 2 diabetes mellitus retarded the angiogenic behavior of MSCs (23).

Recent studies argued that GSK-3β/β-catenin signaling pathway promotes angiogenesis. For example, Yu et al. reported that liraglutide promoted angiogenic ability of endothelial cells by stimulating the production of VEGF, inhibiting the expression of miR-29b-3p, and upregulating GSK-3β/β-catenin pathway (24).

Our study demonstrated that the same type of cells in different states can exert opposite functions on peripheral cells by secreting EVs containing different bioactive molecules. EVs are membrane-enveloped particles that can be released by almost all cell types under physiological or pathological conditions. Thanks to the protection of vesicles, these bioactive molecules are protected from degradation in the extracellular microenvironment (25), so that they can be taken up by receptor cells to regulate the gene expression and protein synthesis in receptor cells. Their biological roles depend on original cell types as well as the microenvironment where cells live. In recent years, EV-mediated cell-cell communication has drawn an increasing attention in regenerative medicine. An increasing amount of research effort has been focused on application of EV-based drugs in clinic. Besides, there are also clinical trials centered on EV as therapeutic tools or delivery drugs. With in-depth understanding of EVs as well as technological progress, EV-based drugs may become a potential therapeutic intervention target for treatment of diseases (26).

## Conclusion

In this study, we demonstrate that the function of EVs secreted from HSFs is changed by high glucose microenvironment. HG-EVs damage the function of HUVECs, which may be partly responsible for impaired angiogenesis in diabetics. In the subsequent study, the different information carried in HG-EVs and NG-EVs will be illustrated.

## Data Availability

The original contributions presented in the study are included in the article/Supplementary Material, further inquiries can be directed to the corresponding author/s.

## References

[1] Liang ZH Pan NF Lin SS Qiu ZY Liang P Wang J Exosomes from mmu_circ_0001052-modified adipose-derived stem cells promote angiogenesis of DFU via miR-106a-5p and FGF4/p38MAPK pathway. Stem Cell Res Ther. (2022) 13:336. 10.1186/s13287-022-03015-7 35870977 PMC9308214

[2] Okonkwo UA DiPietro LA. Diabetes and wound angiogenesis. Int J Mol Sci. (2017) 18:1419. 10.3390/ijms18071419 28671607 PMC5535911

[3] Yu H Peng J Xu Y Chang J Li H. Bioglass activated skin tissue engineering constructs for wound healing. ACS Appl Mater Interfaces. (2016) 8:703–15. 10.1021/acsami.5b09853 26684719

[4] Bian X Ma K Zhang C Fu X. Therapeutic angiogenesis using stem cell-derived extracellular vesicles: an emerging approach for treatment of ischemic diseases. Stem Cell Res Ther. (2019) 10:158. 10.1186/s13287-019-1276-z 31159859 PMC6545721

[5] Wei Q Wang Y Ma K Li Q Li B Hu W Extracellular vesicles from human umbilical cord mesenchymal stem cells facilitate diabetic wound healing through MiR-17-5p-mediated enhancement of angiogenesis. Stem Cell Rev Rep. (2022) 18:1025–40. 10.1007/s12015-021-10176-0 33942217

[6] Zhang Y Su J Ma K Li H Fu X Zhang C. Photobiomodulation promotes hair regeneration in injured skin by enhancing migration and exosome secretion of dermal papilla cells. Wound Repair Regen. (2022) 30:245–57. 10.1111/wrr.12989 34921570

[7] Muralidharan-Chari V Clancy JW Sedgwick A D'Souza-Schorey C. Microvesicles: mediators of extracellular communication during cancer progression. J Cell Sci. (2010) 123:1603–11. 10.1242/jcs.064386 20445011 PMC2864708

[8] Li B Bian X Hu W Wang X Li Q Wang F Regenerative and protective effects of calcium silicate on senescent fibroblasts induced by high glucose. Wound Repair Regen. (2020) 28:315–25. 10.1111/wrr.12794 31943524

[9] Li M Zhao Y Hao H Dong L Liu J Han W Umbilical cord-derived mesenchymal stromal cell-conditioned medium exerts in vitro antiaging effects in human fibroblasts. Cytotherapy. (2017) 19:371–83. 10.1016/j.jcyt.2016.12.001 28081982

[10] Han X Wu P Li L Sahal HM Ji C Zhang J Exosomes derived from autologous dermal fibroblasts promote diabetic cutaneous wound healing through the akt/beta-catenin pathway. Cell Cycle. (2021) 20:616–29. 10.1080/15384101.2021.1894813 33685347 PMC8018430

[11] Bozkurt AS Kaplan DS Ceribasi AO Orkmez M Canak A Tarakcioglu M. An investigation of the effect of extracellular vesicles isolated from mouse embryonic fibroblasts on wound healing in an experimental diabetic mouse model. An Acad Bras Cienc. (2022) 94:e20201562. 10.1590/0001-3765202120201562 35107516

[12] Gangadaran P Oh EJ Rajendran RL Kim HM Oh JM Kwak S Identification of angiogenic cargoes in human fibroblasts-derived extracellular vesicles and induction of wound healing. Pharmaceuticals. (2022) 15(6):702. 10.3390/ph15060702 35745621 PMC9230817

[13] Bian X Li B Yang J Ma K Sun M Zhang C Regenerative and protective effects of dMSC-sEVs on high-glucose-induced senescent fibroblasts by suppressing RAGE pathway and activating smad pathway. Stem Cell Res Ther. (2020) 11:166. 10.1186/s13287-020-01681-z 32349787 PMC7191792

[14] Liang Y Duan L Lu J Xia J. Engineering exosomes for targeted drug delivery. Theranostics. (2021) 11:3183–95. 10.7150/thno.52570 33537081 PMC7847680

[15] Yu M Huang J Zhu T Lu J Liu J Li X Liraglutide-loaded PLGA/gelatin electrospun nanofibrous mats promote angiogenesis to accelerate diabetic wound healing via the modulation of miR-29b-3p. Biomater Sci. (2020) 8:4225–38. 10.1039/D0BM00442A 32578587

[16] Rai V Moellmer R Agrawal DK. Stem cells and angiogenesis: implications and limitations in enhancing chronic diabetic foot ulcer healing. Cells. (2022) 11:2287. 10.3390/cells11152287. 35892584 PMC9330772

[17] Okonkwo UA Chen L Ma D Haywood VA Barakat M Urao N Compromised angiogenesis and vascular integrity in impaired diabetic wound healing. PLoS One. (2020) 15:e0231962. 10.1371/journal.pone.0231962 32324828 PMC7179900

[18] Rezabakhsh A Nabat E Yousefi M Montazersaheb S Cheraghi O Mehdizadeh A Endothelial cells’ biophysical, biochemical, and chromosomal aberrancies in high-glucose condition within the diabetic range. Cell Biochem Funct. (2017) 35:83–97. 10.1002/cbf.3251 28211084

[19] Khaksar M Sayyari M Rezaie J Pouyafar A Montazersaheb S Rahbarghazi R. High glucose condition limited the angiogenic/cardiogenic capacity of murine cardiac progenitor cells in in vitro and in vivo milieu. Cell Biochem Funct. (2018) 36:346–56. 10.1002/cbf.3354 30051492

[20] Alikhani Z Alikhani M Boyd CM Nagao K Trackman PC Graves DT. Advanced glycation end products enhance expression of pro-apoptotic genes and stimulate fibroblast apoptosis through cytoplasmic and mitochondrial pathways. J Biol Chem. (2005) 280:12087–95. 10.1074/jbc.M406313200 15590648

[21] Newman AC Nakatsu MN Chou W Gershon PD Hughes CC. The requirement for fibroblasts in angiogenesis: fibroblast-derived matrix proteins are essential for endothelial cell lumen formation. Mol Biol Cell. (2011) 22:3791–800. 10.1091/mbc.e11-05-0393 21865599 PMC3192859

[22] Zhang P Li Y Guo R Zang W. Salidroside protects against advanced glycation end products-induced vascular endothelial dysfunction. Med Sci Monit. (2018) 24:2420–8. 10.12659/MSM.906064 29679467 PMC5930974

[23] Rezaie J Mehranjani MS Rahbarghazi R Shariatzadeh MA. Angiogenic and restorative abilities of human mesenchymal stem cells were reduced following treatment with serum from diabetes mellitus type 2 patients. J Cell Biochem. (2018) 119:524–35. 10.1002/jcb.26211 28608561

[24] Demelius L Kreiner K Hayn D Nitzlnader M Schreier G. Encoding of numerical data for privacy-preserving record linkage. Stud Health Technol Inform. (2020) 271:23–30. 10.3390/cells11152287 32578537

[25] Wang Y Cao Z Wei Q Ma K Hu W Huang Q VH298-loaded extracellular vesicles released from gelatin methacryloyl hydrogel facilitate diabetic wound healing by HIF-1alpha-mediated enhancement of angiogenesis. Acta Biomater. (2022) 147:342–55. 10.1016/j.actbio.2022.05.018 35580827

[26] Huang Y Kanada M Ye J Deng Y He Q Lei Z Exosome-mediated remodeling of the tumor microenvironment: from local to distant intercellular communication. Cancer Lett. (2022) 543:215796. 10.1016/j.canlet.2022.215796 35728740

